# Distal Femur Allograft Prosthetic Composite Reconstruction for Short Proximal Femur Segments following Tumor Resection

**DOI:** 10.1155/2013/397456

**Published:** 2013-11-17

**Authors:** Bryan S. Moon, Nathan F. Gilbert, Christopher P. Cannon, Patrick P. Lin, Valerae O. Lewis

**Affiliations:** ^1^MD Anderson Cancer Center, Department of Orthopaedic Oncology, P.O. Box 301402, Houston, TX 77230-1402, USA; ^2^Greater Dallas Orthopaedics, 12230 Coit Road, Suite 100, Dallas, TX 75251, USA; ^3^Polyclinic Department of Orthopaedic Surgery, 1001 Broadway, Suite 109, Seattle, WA 98122, USA

## Abstract

Short metaphyseal segments remaining after distal femoral tumor resection pose a unique challenge. Limb sparing options include a short stemmed modular prosthesis, total endoprosthetic replacement, cross-pin fixation to a custom implant, and allograft prosthetic composite reconstruction (APC). A series of patients with APC reconstruction were evaluated to determine functional and radiologic outcome and complication rates. Twelve patients were retrospectively identified who had a distal femoral APC reconstruction between 1994 and 2007 to salvage an extremity with a segment of remaining bone that was less than 20 centimeters in length. Seventeen APC reconstructions were performed in twelve patients. Eight were primary procedures and nine were revision procedures. Average f/u was 89 months. Twelve APC reconstructions (71%) united and five (29%) were persistent nonunions. At most recent followup 10 patients (83%) had a healed APC which allowed WBAT. One pt (8%) had an amputation and one pt (8%) died prior to union. Average time to union was 19 months. Four pts (33%) or five APC reconstructions (29%) required further surgery to obtain a united reconstruction. Although Distal Femoral APC reconstruction has a high complication rate, a stable reconstruction was obtained in 83% of patients.

## 1. Introduction

Resection of large skeletal tumors can result in short metaphyseal juxtaarticular segments of host bone which can pose a reconstructive challenge to the musculoskeletal tumor surgeon. In addition, aseptic loosening or fracture around a standard reconstruction can lead to loss of bone stock so that only a short metaphyseal segment of host bone remains for fixation in revision surgery. Limb salvage reconstructive options in this scenario include the use of a standard endoprosthesis with fixation of the stem into the short segment of host bone, use of custom implants allowing for cross-pin fixation of the endoprosthesis to the host bone, use of an endoprosthesis to replace the entire bone, and use of a composite of an allograft and an endoprosthesis [1–3].

**Figure 1:**
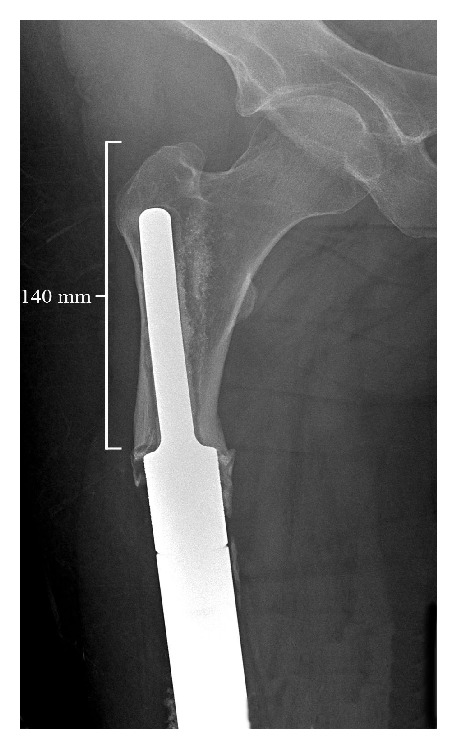
Short stemmed cemented endoprosthesis with aseptic loosening.

**Figure 2:**
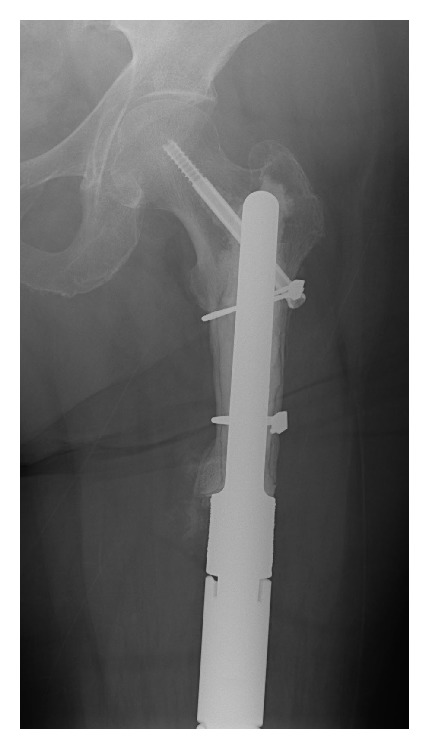
Customized cemented stem with screw fixation.

**Figure 3:**
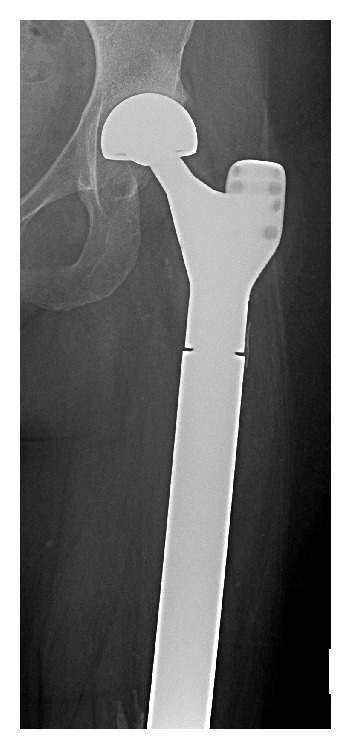
Total femur endoprosthesis.

**Figure 4:**
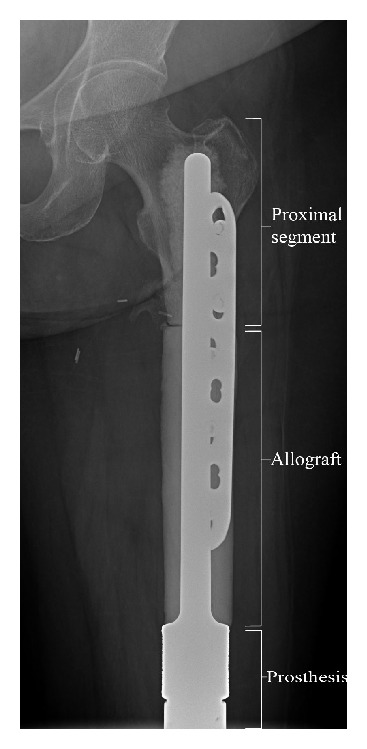
Distal femur APC.

**Figure 5:**
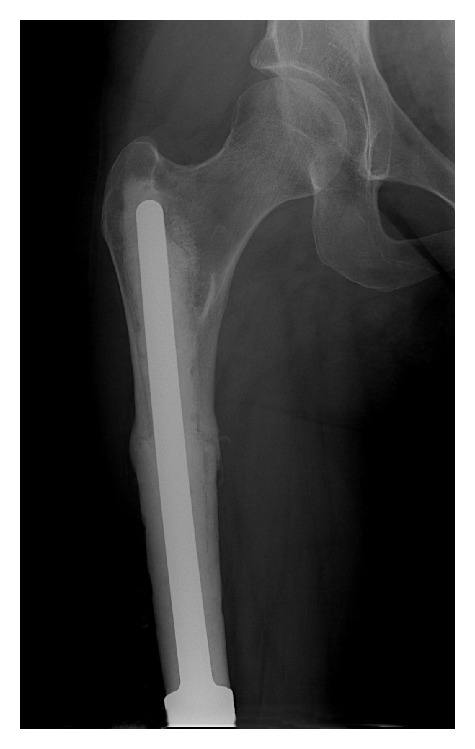
Healed distal femur APC.

Use of a standard, modular endoprosthesis with cement or press fit fixation in this setting has not been directly investigated to our knowledge; however, the use of a short stem cemented into the metaphyseal segment would be expected to have a high rate of aseptic loosening due to the high stress imparted on the relatively short interface between host bone and cement [2] (Figure 1). Use of cross-pin fixation of a custom prosthesis to host bone has been described previously with a low rate of aseptic loosening [1] (Figure 2). This technique is limited by decreasing intraoperative flexibility and adding extra time and cost. Replacement of the entire bone with an endoprosthesis is also a described technique that has the advantage of early weight bearing, but it has the disadvantage of increased rehabilitation and higher rates of joint dislocations [3–5] (Figure 3). Allograft prosthetic composite (APC) reconstruction restores bone stock which allows the use of a longer stem, increases intraoperative flexibility, and provides the durability of an endoprosthesis (Figures 4 and 5). Figures 1–4 demonstrate the main types of reconstruction currently used for short juxtaarticular metaphyseal segments. To the best of our knowledge, no series has previously reported on APC reconstruction for short metaphyseal segments of host bone. 

Our purposes were to determine (1) the functionality of these patients, union rates, rates of reoperation, and other complications that may occur with this type of reconstruction and (2) if this type of reconstruction is an acceptable alternative to other reconstructive options.

## 2. Methods

We retrospectively reviewed 12 patients who underwent allograft prosthetic composite reconstruction after resection of large skeletal tumors between 1994 and 2007. All patients had 20 cm or less of host bone remaining. Average age of the patients was 19 years (range, 7–56 years). Five males and seven females comprised the study group. Diagnosis was osteosarcoma in 10 patients, chondrosarcoma in 1 patient, and giant cell tumor in 1 patient. Location of the tumor was the distal femur in all 12 patients. Six patients received neoadjuvant and adjuvant chemotherapy, one received neoadjuvant chemotherapy, one received adjuvant chemotherapy, and four received no chemotherapy in the perioperative period. No patients received radiation therapy. 

A total of seventeen allograft prosthetic reconstructions were performed in these twelve patients. Eight reconstructions were performed as primary limb salvage procedures and nine were performed as revision procedures.

 At the time of surgery, all patients had an extensile surgical approach to expose the area of interest. Freeze-dried allografts were used in all cases. The allografts were thawed in antibiotic solution and then prepared on a back table by cutting the allograft to required length and reaming to appropriate diameter. Transverse osteotomies were utilized and were manipulated as needed to maximize allograft-host bone contact. Cement fixation was used in all cases to fix the stem in both the allograft and host bone. A Biomet (Warsaw, IN) custom expandable implant was used in 5 patients, a Biomet long-stem modular rotating hinge total knee replacement was used in 3 patients, a Biomet modular distal femur endoprosthesis was used in one patient, a Stryker (Mahwah, NJ) modular distal femur endoprosthesis was used in one patient, and a Wright Medical (Memphis, TN) noninvasive custom expandable endoprosthesis was used in two patients. Additional plate fixation was used in 7 patients. Supplemental autograft, allograft, or demineralized bone matrix was used at the host-allograft interface in all but one patient. Postoperative rehabilitation involved early range of motion and touchdown weight bearing until there was radiographic evidence of healing. 

The radiographs and medical records of these patients were analyzed to determine functional, radiographic, and clinical outcome. The remaining host bone segments were measured from the tip of the greater trochanter to the osteotomy site. Radiographs were also analyzed to determine union, which was defined as osseous bridging of at least 3 of 4 cortices at the osteotomy site. 

## 3. Results

Average duration of followup from the date of the original surgery was 89 months (range, 31–177 months). The average length of bone remaining after resection of the tumor was 13 cm (range, 7–20 cm). One patient died of disease prior to union, one died after union occurred, and one patient underwent an amputation. Three patients were lost to followup at 133, 177, and 78 months.

The twelve patients underwent seventeen APC reconstructions (Table 1). The five extra reconstructions in three patients were performed for three nonunions and two prosthesis failures. Primary union was achieved in eight reconstructions (47%), and secondary union was achieved in four reconstructions (24%) via secondary bone grafting. Five reconstructions (29%) were persistent nonunions. Therefore, bony union was achieved in 12 reconstructions (71%) or ten patients (83%) at an average of nineteen months (range, 4–29 months). Of these ten patients, five (50%) required further surgery to obtain union.

Graft related complications including nonunion requiring bone grafting, persistent nonunion requiring APC revision, allograft fracture, infection, and stem perforation occurred in eight patients (67%) (Table 1). Three patients (25%) underwent five complete APC revisions. Three were for persistent nonunions, one was for a broken stem, and one was for stem perforation through the allograft. The average time to revision of the reconstruction in these patients was 29 months (range, 11–48 months). There were two deep infections. One was a fungal infection that was successfully treated with debridement and antifungals. The second was a staph aureus infection that eventually ended in amputation. A third patient made telephone contact 60 months after surgery with concerns of an infection, but the patient did not return for evaluation and was lost to followup. 

In terms of functional outcome, all ten patients with united reconstructions were weight bearing as tolerated at their most recent follow-up visit. Two of these patients use assistive devices for ambulation. Current MSTS scores were available for six of the ten patients. The average score was 90%. The average time from surgery to evaluation for scoring was 82 months (range, 52–90 months). The MSTS score for the patient that underwent amputation was 63%.

## 4. Discussion

At first glance, the above results may seem discouraging for the use of APC reconstruction in the setting of short metaphyseal bone segments. Certainly, the complication rate is significant, but it should be noted that the complications were manageable in the majority of patients. It should also be noted that once union was achieved, the reconstruction does appear durable in that there has only been one graft related complication following union. Also, the allograft provided sufficient bone stock for a standard revision in the one case of stem loosening. In the case of the broken stem, it was the surgeon's preference to replant an APC rather than attempting stem extraction from the allograft. 

Other limb sparing options are available for this difficult type of reconstruction including cementation of a modular endoprosthesis into the short segment of host bone, resection of the entire bone and replacement with an endoprosthesis, and cross-pin fixation of a custom endoprosthesis to the host bone. All of these approaches have technical challenges, advantages, and disadvantages. Cementing a short stem of a standard modular endoprosthesis into a short metaphyseal segment would be expected to have a high rate of aseptic loosening and implant failure. This has been confirmed in clinical studies that report poor results in lower extremity endoprosthetic reconstruction when addressing bone defects left by larger resections [2, 6]. In addition, biomechanical studies using a canine model have shown that cement fixation of endoprostheses alone leads to a more compliant reconstruction when dealing with large femoral defects [7, 8].

Several techniques have been devised to overcome the limitations of standard modular endoprostheses in this setting, including total replacement endoprostheses, cross-pin fixation of custom endoprostheses, and use of allograft prosthetic composite reconstruction. Total bone endoprostheses are useful reconstructive devices, but their implementation necessitates the removal of two joints. This creates the possibility of complications and wear debris at two articulations [5]. Rehabilitation of two artificial joints is more rigorous and complicated than one, especially if one of the joints has a significant risk of dislocation. There have been techniques described to limit the risk of dislocation in total femur endoprostheses, but hip joint preservation is favorable for lower extremity function [9]. One advantage of total endoprosthetic replacement is that weightbearing may be allowed right away. In addition, concern for healing the host allograft junction is obviated. Although the numbers of studies on total endoprosthetic replacement are limited, functional outcome has been promising [3, 5].

Cross-pin fixation of a custom prosthesis into the remaining short metaphyseal defect has also been described [1]. Twelve patients with short, metaphyseal segments were included in this study. Cement fixation of the stem into the remaining host bone was used in addition to screws. Only one of the stems used for short metaphyseal articular segments failed, and reconstructive success was achieved with a relatively low complication rate. One drawback to this technique is the fact that all implants must be custom made. Therefore, several weeks of manufacturing time is required and the use of a custom implant diminishes intraoperative flexibility. This may prevent their use in more urgent reconstructive scenarios such as displaced pathologic or periprosthetic fracture.

The use of APCs for reconstruction of short metaphyseal segments has not been examined previously. Allograft prosthetic composite reconstruction has been shown to be a viable reconstructive technique for a variety of reconstruction sites including the proximal femur and tibia [10–13]. The advantage of providing local bone stock is combined with intraoperative flexibility and the durability of an endoprosthesis. Allograft prosthetic composites can be used after primary tumor resection or in revision of a failed reconstruction. The main disadvantage is that weight bearing is usually limited until healing at the host-allograft junction occurs [14]. The use of allograft prosthetic composites used for revision of failed distal femur endoprostheses has been described and it was determined that this was a successful option for revising a failed distal femur endoprosthesis, but the length of remaining bone was not separately analyzed [15]. The results of total femur allograft prosthetic composites have also been previously reported [4]. A total femur allograft with a bipolar hemiarthroplasty and total knee arthroplasty was used. Logistically, this is the same as a total replacement endoprosthesis and different from the technique described in our series.

Limitations of this study include small sample size, retrospective design, and patient heterogeneity. Given that the need for distal femoral APC is an uncommon event, small sample size and retrospective design should not be considered a major weakness. Similarly, since neoplasms of the distal femur affect a wide patient demographic, patient heterogeneity is unavoidable. However, it is reasonable to assume that some aspects of heterogeneity such as the use of chemotherapy, expandable versus unexpandable implants, and primary versus revision reconstructions may have an influence on the rate of union. For example, it is notable that four of the five nonunions occurred in patients with expandable prostheses. Although this may represent an interesting trend, our numbers are too small for any meaningful statistical analysis. Finally, three patients were lost to current followup and MSTS scoring. Given that one of these patients underwent four APC reconstructions and one did not follow up after making contact regarding concerns of infection, it is likely that the MSTS scores are skewed in a positive direction. However, these three patients did have an average followup of 129 months and were all ambulating at their final visits.

Distal femur APC reconstruction can be useful in reconstruction of short metaphyseal segments both after primary tumor resection and for salvage of a failed prior reconstruction. The complication rate was high even in patients who went on to have a durable reconstruction; therefore, patients should be counseled to provide reasonable expectations. As the primary union rate was low, early bone grafting should be considered. It is our current practice to consider bone grafting no earlier than 6 months postoperatively. Autograft, allograft chips, and/or demineralized bone matrix may be used and is determined by patient and surgeon preference. Once union is achieved, the patients can be expected to have an acceptable level of function and a durable reconstruction. Further studies are required to determine optimal reconstructive techniques for this challenging scenario.

## Figures and Tables

**Table 1:** 

Patient	Age	Diagnosis	Type	Expandable	Residual femur	Union	Followup	Complications
1	12	OGS	Primary	Yes	14 cm	29 mos	31 months, death	Death
2	15	OGS	Primary Revision Revision Revision	Yes Yes Yes No	20 cm 16 cm 17 cm 17 cm	No 12 mos ? mos 21 mos	133 months, lost	Nonunion Broken stem Stem perforation Bone graft
3	25	OGS	Revision	No	17 cm	29 mos	177 months, lost	Bone graft/plating X2
4	8	OGS	Primary	Yes	10 cm	26 mos	78 months, lost	Bone graft, stem loosening ? infection
5	8	OGS	Primary Revision	Yes Yes	7 cm 7 cm	No No	118 months, current	Nonunion Nonunion, infection, amp
6	9	OGS	Primary	Yes	10 cm	No	31 months, death	Nonunion, death
7	9	OGS	Revision	No No	17 cm 17 cm	No 12 mos	168 months, current	Fracture, nonunion None
8	7	OGS	Primary	Yes	11 cm	22 mos	83 months, current	Fungal infection
9	31	OGS	Revision	No	13 cm	21 mos	81 months, current	None
10	56	CHSA	Primary	No	10 cm	28 mos	55 months, current	Bone graft
11	35	GCT	Revision	No	11 cm	10 mos	60 months, current	None
12	8	OGS	Primary	Yes	10 cm	4 mos	52 months, current	Tibia fracture
